# Late-Onset Neutropenia in Clozapine Users: Unrelated or Drug-Induced? A Case-Registry Analysis of Incidence, Characteristics, and Rechallenge Attempts

**DOI:** 10.1093/schbul/sbaf148

**Published:** 2025-08-26

**Authors:** Aviv Segev, Risha Govind, Ebenezer Oloyede, Cecilia Casetta, Megan Pritchard, Amelia Jewell, Matthew Broadbent, Harsimran Kaur Makan, David Taylor, James MacCabe

**Affiliations:** Department of Psychosis Studies, Institute of Psychiatry, Psychology and Neuroscience, King’s College London, London, SE5 8AF, United Kingdom; NIHR Biomedical Research Centre for Mental Health South London and Maudsley NHS, London, SE5 8AF, United Kingdom; School of Medicine, Faculty of Medical and Health Sciences, Tel Aviv University, Tel Aviv, 69978, Israel; Shalvata Mental Health Centre, Hod Hasharon, 4534708, Israel; Department of Psychosis Studies, Institute of Psychiatry, Psychology and Neuroscience, King’s College London, London, SE5 8AF, United Kingdom; NIHR Biomedical Research Centre for Mental Health South London and Maudsley NHS, London, SE5 8AF, United Kingdom; Pharmacy Department, South London and Maudsley NHS Foundation Trust, London, SE5 8AZ, United Kingdom; Department of Psychiatry, University of Oxford, Oxford, OX3 7JX, United Kingdom; Department of Psychosis Studies, Institute of Psychiatry, Psychology and Neuroscience, King’s College London, London, SE5 8AF, United Kingdom; National Psychosis Service, South London and Maudsley NHS Foundation Trust, London, BR3 3BX, United Kingdom; NIHR Biomedical Research Centre for Mental Health South London and Maudsley NHS, London, SE5 8AF, United Kingdom; NIHR Biomedical Research Centre for Mental Health South London and Maudsley NHS, London, SE5 8AF, United Kingdom; NIHR Biomedical Research Centre for Mental Health South London and Maudsley NHS, London, SE5 8AF, United Kingdom; Pharmacy Department, South London and Maudsley NHS Foundation Trust, London, SE5 8AZ, United Kingdom; Department of Psychosis Studies, Institute of Psychiatry, Psychology and Neuroscience, King’s College London, London, SE5 8AF, United Kingdom; NIHR Biomedical Research Centre for Mental Health South London and Maudsley NHS, London, SE5 8AF, United Kingdom; National Psychosis Service, South London and Maudsley NHS Foundation Trust, London, BR3 3BX, United Kingdom

**Keywords:** schizophrenia, blood dyscrasia, treatment resistance, adherence

## Abstract

**Background and Hypothesis:**

Clozapine treatment carries a risk of blood dyscrasias (BD) and requires indefinite monitoring in many jurisdictions, a major factor in its under-utilization. Although previous studies suggest BD risk is highest early in treatment, BD events have also been reported after many years. This study compares early vs late (>6 months) suspected blood dyscrasias (SBD) and examines rechallenge outcomes as a marker for clozapine-related causation.

**Study Design:**

A retrospective analysis of electronic health records from a large UK mental health service gathered demographic data, characteristics of SBD events, and outcomes of clozapine rechallenge, defined as reinitiation after SBD-related discontinuation. These variables were compared between early- and late-onset SBD groups using a 6-month treatment duration cutoff.

**Study Results:**

Of 130 patients with SBD leading to clozapine cessation, 59 had early-onset SBD. The incidence rate before 6 months was 5.54% per year vs 0.53% after 6 months, reflecting an incidence rate ratio of 10.4. Early-onset patients were younger, received lower clozapine doses, and had fewer concurrent antipsychotics. Of 81 rechallenge attempts, 71 (87.7%) were successful, with a mean follow-up of 2.5 years. No significant differences in characteristics or rechallenge outcomes were found between the early- and late-onset groups.

**Conclusions:**

Though less frequent, late-onset SBD shares similar characteristics with early-onset SBD and has a comparable risk of recurrence on clozapine rechallenge. Vast majority of clozapine rechallenges are successful, including early-onset BD, suggesting they are not clozapine-induced. However, clozapine-induced BD, defined by recurrence upon rechallenge, may rarely occur even after years of treatment.

## Introduction

Around 20% of patients with schizophrenia fail to respond to 2 or more adequate antipsychotic trials,^1^ a clinical situation referred to as treatment-resistant schizophrenia (TRS).^2^ The only evidence-based treatment for TRS is clozapine,^3^ with a 60%-70% response rate.^4^

Despite its advantages,^5^ clozapine remains underused.^6–9^ Adverse effects, particularly clozapine-induced blood dyscrasias (BD), are a major deterrent.^10^^,^^11^ Most developed countries manage this rare but serious risk of BD through mandatory blood monitoring protocols, which vary internationally.^12^^,^^13^ In the United Kingdom, monitoring is weekly for 18 weeks, biweekly for the next 34 weeks, then monthly.^12^^,^^13^ Studies have shown that this burdensome monitoring discourages initiation and contributes to non-adherence.^11^^,^^14^^,^^15^

Neutropenia occurs in about 3% of clozapine users, with a risk of agranulocytosis of less than 1%.^16–18^ A recent meta-analysis showed that 75% of mild neutropenia cases do not progress, and fatalities from agranulocytosis are rare.^17^ Recent studies have suggested that many cases of neutropenia are not true BD but reflect either an incidental finding or non-clozapine etiology, such as Benign Ethnic Neutropenia (BEN), viral infections, or primary hematological disorder.^19–21^ Furthermore, specific patterns have been identified that distinguish true clozapine-induced BD.^21^ Therefore, many BD events are merely suspected blood dyscrasias (SBD) and do not represent clozapine-induced neutropenia.

Despite this reassuring data, psychiatrists often remain concerned,^14^ a reluctance sometimes labeled as “clozaphobia”.^22^ Psychiatrists are even more cautious about attempting a clozapine rechallenge, despite evidence suggesting that this can often be successfully achieved.^23–26^ As a result, many patients with TRS are denied further access to the only drug likely to help them.

The pathophysiology of clozapine-induced BD remains unclear.^27^ Evidence suggests a bioactivation of clozapine metabolites, generating nitrenium ions, inducing an autoimmune response.^28^ This supports the observation that BDs occur most often early in treatment.^29^ An autoimmune model also aligns with genetic associations of Human Leukocyte Antigen (HLA) variants with BD risk.^30^ However, different mechanisms may underlie BD events across patients, influencing timing and risk.

Other factors have been linked to SBD during clozapine treatment, but their role in early vs late SBD is unclear. One such factor is concomitant valproate use.^31^^,^^32^ Another is BEN, common among individuals of African descent and characterized by lower baseline neutrophil counts due to genetic factors. Though BEN is not associated with a higher risk of agranulocytosis, undiagnosed BEN may lead to inappropriate clozapine discontinuation.^10^ Modified monitoring criteria have therefore been established for BEN patients to avoid unnecessary clozapine interruption.

Probably one of the most important factors to consider when examining SBD is temporality: time since clozapine initiation. The risk of SBD is at its highest at the initial period of treatment,^16–18^^,^^33^ yet several papers described late-onset SBD occurring even after many years of treatment.^34–37^ Systematic data examining the differences between early- and late-onset BD is sparse.^24^ It is unclear whether they share the same pathophysiology or require different clinical management.

Given the burden of lifelong monitoring^13^ and its deterrent effect on clozapine use, it has been suggested to relax the monitoring protocols after extended treatment duration.^37–40^ A major challenge is distinguishing the “true” clozapine-induced BD vs incidental or non-clozapine-induced ones. As the mechanisms remain unknown, there are 2 ways to ascertain causality with reasonable certainty—either establishing a definite alternative cause, which is rarely straightforward, or by rechallenging the patient with clozapine. Recurrence of BD, presuming other causative factors have been identified and removed, can serve as a reliable proxy to differentiate between “true” clozapine-induced BD and BD due to other causes.

This study aims to characterize potential differences between early and late BD and to compare the success rate of clozapine rechallenge in both groups. This may provide deeper insight into the possible differences between these groups and contribute to future clinical guidelines for managing clozapine-induced BD.

## Methods

To identify patients developing SBD during clozapine treatment, we investigated data of patients registered to the South London and Maudsley (SLaM) National Health Service (NHS) Foundation Trust. South London and Maudsley provides almost all secondary mental health services for a catchment area of 1.3 million people residing in South London, being one of the largest mental health organizations in Europe. Patients’ data was collected from the Clinical Research Interactive Search (CRIS), an anonymized database which contains the entire clinical records of SLaM patients from 2007 to date, designed for research purposes, and is fully described elsewhere.^41^ The CRIS database is linked to several external databases, among them is the national Zaponex Treatment Access System (ZTAS), which serves as the clozapine registry in SLaM, and to the Clozapine Non-Rechallengeable Database (CNRD), which contains patients who developed SBD that necessitated clozapine cessation.

The term “SBD” was used instead of simply “blood dyscrasia” to reflect the clinical uncertainty surrounding many of these events. While “blood dyscrasia” refers broadly to any imbalance in blood components, transient changes in blood counts are common and can result from benign or subclinical conditions such as mild infections. The use of “SBD” was intended to distinguish between these non-pathological variations and those more likely to be clozapine-induced hematological disorders.

### Study Sample

The study sample (in part described in an additional study^42^) included all patients in the CRIS database from its establishment in 2007 who were also registered in ZTAS and the CNRD up to October 2019. This information was then confirmed by manual inspection of the patients’ electronic health record (anonymized by CRIS, as detailed) by a senior psychiatrist (A.S.). Patient demographics and clinical data were obtained from both structured fields and clinical notes within CRIS. To establish accurate start and stop dates of clozapine, we developed an algorithm, described in detail elsewhere.^43^

### Ethics Approval

Ethical approval for the use of CRIS as a research dataset was given by Oxfordshire Research Ethics Committee C (23/SC/0257) and the CRIS oversight committee granted permission for this study.

### Clozapine Administration

Clozapine initiation was defined as the day when clozapine was first dispensed. If a patient had more than 1 trial of clozapine more than 1 month apart, the most recent initiation prior to the SBD was defined as the relevant clozapine trial. The study endpoint was defined as January 1, 2020, discharge from SLaM services or death, whichever came first.

### Blood Dyscrasia

Abnormal results are defined by the UK Medicines and Healthcare Products Regulatory Agency (MHRA) guidelines, which set the cutoff for blood dyscrasia, using a traffic light system. A “red result” is defined either by total leucocytes absolute number <3000 cells per microliter of blood, or total neutrophil absolute number <1500/μL. An “Amber result” is defined either by total leucocyte absolute number between 4000 and 3000/μL or total neutrophil absolute number between 2000 and 1500/μL. In this paper, we used the MHRA definition of neutropenia (<1500/μL), as this threshold is currently sufficient to trigger clinical management that may lead to clozapine cessation, as is the case in other countries.^13^ The MHRA also permits a relaxation of these thresholds for patients diagnosed with BEN, in which the cut-off is 500 cells/μL lower (ie, a patient diagnosed with BEN would be considered to have a red result only if their neutrophil count were to be less than 1000/μL instead of 1500/μL). As previously discussed in the literature, the term “BEN” is both imprecise and carries problematic sociocultural implications. Ideally, the more biologically accurate term, “Duffy antigen receptor for chemokines (DARC)—null”, would be used.^44^ However, the databases employed in this study record BEN as a diagnostic field, and Duffy antigen testing was not available for the included patients. The diagnosis was typically made based on persistently low neutrophil counts in patients from specific ethnic backgrounds. For these reasons, the term BEN is used in this paper, despite its recognized limitations. In the United Kingdom, there are 3 manufacturers of clozapine, each running its own monitoring system, and all operating in accordance with the MHRA regulations. The one used at SLaM NHS foundation trust is the national ZTAS clozapine register, which is linked to the CRIS system, and contains the details of every patient in SLaM who has ever been treated with clozapine, including laboratory data and the history and current clozapine status.

### Clozapine Non-rechallengeable Status and Rechallenge

Clozapine non-rechallengeable (CNR) status is determined according to the MHRA guidelines, that is, 2 consecutive “red results”. However, the medical director of ZTAS can assign a CNR status in two additional scenarios in which it is considered unsafe to expose the patient to clozapine: either repeated mild neutropenic events not including 2 “reds” (meaning, a series of low amber or alternating red and amber results), or when a single “red result” is not followed up by a repeat full blood count. On the other hand, the manufacturer’s medical director can authorize agreements on a “named patient” basis (referred to as “off-license”) to patients with special hematological circumstances, such as patients receiving chemotherapy or immunosuppressants. In these cases, specific thresholds are set. To note, in previous years, CNR status was assigned to patients even in cases of a single red result, some of whom were still listed on the CNRD. These patients were excluded from the study.

Rechallenge was defined as any supply of clozapine by a patient previously assigned a CNR status. Successful rechallenge was defined as no recurrence of ZTAS assignment of CNR status, until the study endpoint or clozapine cessation not resulting from a return to CNR status.

### Statistical Analysis

Descriptive statistics were used to summarize patient demographics, clinical characteristics, and outcomes. Continuous variables were compared using independent samples *t*-tests if normally distributed, or Mann–Whitney *U* tests if distributions were non-normal. Categorical variables were compared using Chi-square tests; Fisher’s exact test was applied when expected cell counts were below 5. For categorical variables with more than 2 groups and low cell sizes, Monte Carlo simulation was used to estimate significance. Incidence rates of BD events were calculated based on the duration of clozapine exposure in early and late groups. Incidence rate ratios (IRRs) were calculated by comparing these rates between early and late onset groups. To adjust for the unequal rechallenge rates between groups, the observed rechallenge failure rate was extrapolated to the non-rechallenged group to estimate the adjusted number of likely clozapine-induced BD events in each group. To address potential selection bias in rechallenge attempts, a worst-case bounds analysis was conducted by modeling extreme scenarios: (1) assuming all non-rechallenged cases were clozapine-induced BD, and (2) assuming none were. Intermediate estimates were also calculated under moderate assumptions. All statistical analyses were conducted using IBM Statistical Package for the Social Sciences (SPSS) version 29, with significance set at *P* < .05 (two-tailed).

## Results

**Figure 1:**
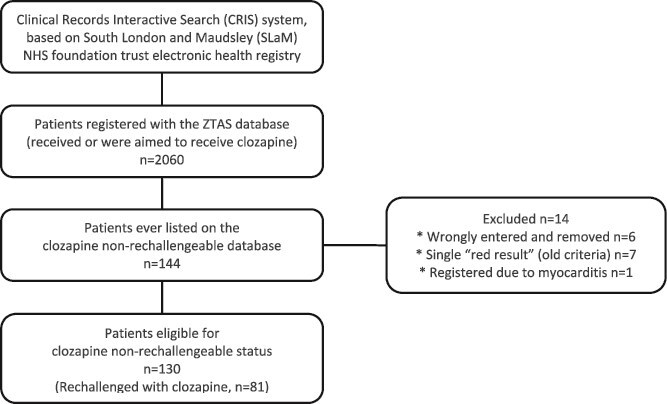
Sample Selection Flowchart

One hundred forty-four cases of CNR status were identified, out of 2060 patients registered as ever taking clozapine. Of those, 14 were excluded as they did not fulfill formal criteria to be assigned with CNR status. The sample selection is illustrated in Figure 1.

### BD Events

**Figure 2:**
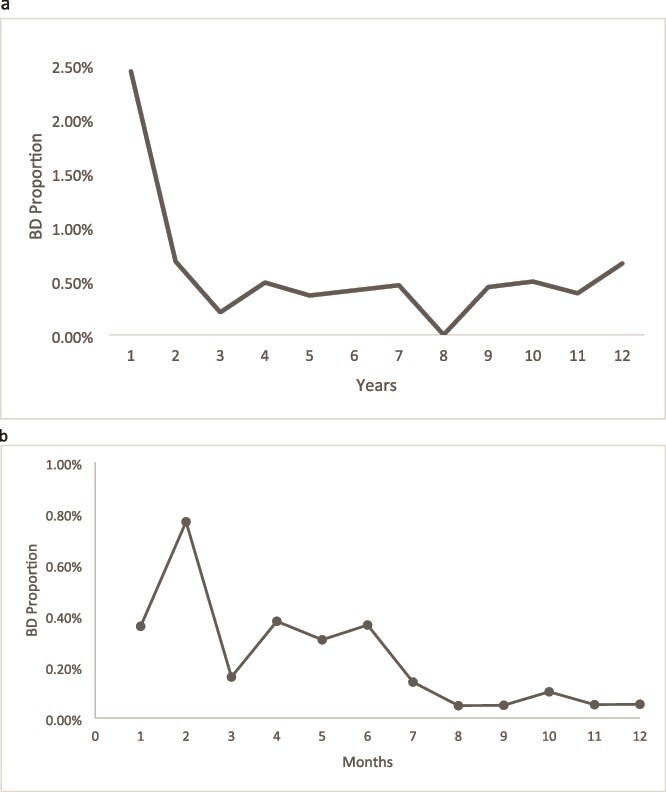
a. Proportion of Clozapine Users Developing Suspected Blood Dyscrasia (SBD) Over Time (By Years) b. Proportion of Clozapine Users Developing SBD during the First Year (By Months)

Clozapine non-rechallengeable events spread from 1 to 10 046 days (27.5 years) after clozapine initiation (median 281.5 days, IQR 2164). The number of events decreased sharply over time: 68 (52.3%) of cases occurred during the first year, 12 (9.2%) during the second year, and the remaining 50 cases (38.5%) spread across years 3-28 post clozapine initiation. The rate, calculated by the number of patients developing CNRD in each year relative to the entire patient population that had received clozapine for at least that duration, decreased sharply (Figure 2a). As the incidence rate in the first year was substantially higher, a breakdown of the first year by months is illustrated (Figure 2b).

There was a sharp decline in the number of cases after 6 months, 86.8% of cases occurring within the first year were within the first 6 months, accounting for 45.4% of all CNR cases. Thus, early SBD was defined as occurring within the first 6 months of treatment. A comparison of patients with early vs late SBD is presented in Table 1. Based on the duration of each clozapine trial, the incidence rate of SBD was 5.54% per year before 6 months, compared to 0.53% per year after 6 months, reflecting an IRR of 10.4. As previous literature has suggested an increased risk during various timeframes (eg, the first year, first 2 years), a detailed breakdown of events by time period is provided in the supplementary material (Table S1).

**Table 1:** Comparison of Patients with Early vs Late Onset Suspected Blood Dyscrasias

	Early SBD *n* = 59 (45.4%)	Late SBD *n* = 71 (54.6%)	t/χ^2^	*P* value
Demographics				
Age at CNR event (mean, SD)	35.0 (14.6)	43.1 (12.6)	t(120) = 3.416	.001
Age at clozapine initiation (mean, SD)	34.0 (14.6)	35.2 (10.8)	t(105) = 0.548	.59
Gender (*n*, % male)	38 (64.4%)	44 (62.0%)	χ^2^(1) = 0.082	.77
Ethnicity (*n*, % White, *n*, % Black)	22 (37.9%), 26 (44.8%)	37 (52.1%), 27 (38.0%)	χ^2^(2) = 1.764	.42
BEN diagnosis prior to CNR event (*n*, %)	3 (5.2%)	3 (4.3%)	χ^2^(1) = 0.056	.57
CNR event				
Time to CNR event, days (mean, SD)	74.2 (51.5)	2698.2 (2318.4)	t(70) = 9.466	.001
Clozapine dose at CNR event (mean, SD)	298.9 (126.2)	365.5 (140.8)	t(120) = 2.731	.007
Leukopenia (*n*, %)	24 (40.7%)	25 (37.3%)	χ^2^(1) = 0.149	.70
Neutropenia (*n*, %)	54 (91.5%)	59 (86.8%)	χ^2^(1) = 0.730	39
Lymphopenia (*n*, %)	9 (15.3%)	11 (16.2%)	χ^2^(1) = 0.020	.89
Agranulocytosis (*n*, %)	4 (6.8%)	3 (4.2%)	χ^2^(1) = 0.444	.70
Concomitant medications at CNR event				
Concomitant antipsychotic (*n*, %)	6 (10.7%)	20 (30.3%)	χ^2^(1) = 6.932	.008
Concomitant valproate (*n*, %)	20 (35.7%)	31 (47.0%)	χ^2^(1) = 1.578	.21
Concomitant other neutropenia-risk drugs^a^ (*n*, %)	1 (1.9%)	12 (17.9%)	χ^2^(1) = 8.041	.006
Concomitant lithium (*n*, %)	5 (8.9%)	5 (7.6%)	χ^2^(1) = 0.074	.79

a Medications include lamotrigine (5), lamotrigine and gliclazide (1), gliclazide (2), spironolactone (2), carbamazepine (1), and mesalazine (1). Leukopenia, leucocyte count <3000 cells/μL; Neutropenia, neutrophil count <2000 cells/μL; Lymphopenia, lymphocyte count <1000 cells/μL; Agranulocytosis, neutrophil count <500 cell/μL. Abbreviation: CNR, clozapine non-rechallengeable.

### Rechallenge Attempts

Of the 130 patients developing SBD, 81 (62.3%) were rechallenged on clozapine. The mean interval from entry to the CNRD to clozapine rechallenge was 3.2 years (SD = 5.2, min 0.01, max 21.0 years). Rechallenged patients were younger (37.1 years [SD 13.0] vs 43.2 [SD 15.0], *P* = .02) and had longer clozapine treatment until CNR status was assigned (median of 4.7 [IQR 6.1] vs 3.2 [IQR 5.5], *P* = .03). Though rechallenged patients were less likely to have had an early SBD event, the difference was not statistically significant (39.5% vs 55.1%, *P* = .08). The comparison of patients who were rechallenged to those who were not rechallenged is presented in the supplementary material (Table S2).

Of the 81 patients rechallenged on clozapine, 59 (72.8%) continued their clozapine until the study endpoint (the end of the study timeframe, death, or discharge from SLaM psychiatric service), with a mean follow-up of 2.5 years (SD 2.6, median 1.9, IQR 2.8, min 0.1, max 12.8). Of the 22 patients who stopped clozapine treatment prior to the study endpoint, 5 were due to adverse events other than SBD (constipation, seizures, vomiting, rash, cardiac problems), 5 were switched to a different antipsychotic agent due to non-adherence, and 2 had multiple non-consecutive “red” or “amber” results that resulted in clinical decision to stop clozapine due to the difficulties in managing those patients, despite not fulfilling the criteria for CNR status. Ten rechallenged patients (12.3%) had their CNR status reinstated due to repeated BD (unsuccessful rechallenge), with no statistical difference between the groups (early-BD 15.6%, late-BD 10.2%). Of the ten failed rechallenges, recurrent BD occurred within 2 months in 8 cases, while the remaining 2 occurred at 3.2 and 4.6 months. This indicates that the timeframe from clozapine rechallenge to an additional CNR BD event ranged from 26 to 139 days. Interestingly, the initial BD event leading to clozapine cessation was mild in 8 of these 10 cases, moderate in 1, and undocumented in 1. One case had a BEN diagnosis assigned prior to the initial BD event, 2 received the diagnosis afterward, and 7 were not assigned a BEN diagnosis at any point before or after the initial or subsequent BD events.

Fifty (60.5%) of the rechallenged patients were originally assigned a CNR status due to late SBD and 32 (39.5%) due to early SBD (Table 2). Rechallenge was more common in the late SBD group (50 out of 71, 69.0%) than the early SBD group (32 out of 59, 54.2%), though this difference was not statistically significant (*P* = .08). Time to rechallenge was significantly shorter after late SBD rather than early SBD (2.0 vs 5.1 year, respectively, *P* = .009). Concomitant use of antipsychotics was more common in the late SBD group (*P* = .02). In 2 rechallenge cases, clozapine was discontinued due to repeated borderline or fluctuating neutrophil counts, without fulfilling formal CNR criteria. These cases were classified as a separate outcome category (“Borderline outcome”) and are reported in Table 2. A comparison of successful rechallenges vs unsuccessful rechallenges is presented in Table 3. To note, the 2 “borderline outcomes” were excluded from the primary outcome analysis of “successful” vs “unsuccessful” rechallenge, as either classification risked misrepresentation: categorizing them as “successful” would overlook the fact that clozapine was ultimately discontinued due to hematological concerns, while assigning them as “unsuccessful” would be inaccurate, as they did not meet the formal criteria for CNR that defined failed rechallenges in this study. A comparison of successfully rechallenged patients with early vs late SBD is provided in the supplemental material (Table S3).

**Table 2:** Comparison of Early vs Late Suspected Blood Dyscrasias Rechallenged Patients

	Early SBD rechallenge *n* = 32	Late SBD rechallenge *n* = 49	t/χ^2^/U	*P* value
Demographics				
Age at CNR event (mean, SD)	31.3 (12.2)	40.9 (12.2)	t(79) = 3.459	.001^*^^*^
Gender (*n*, % male)	20 (62.5%)	33 (67.3%)	χ^2^(1) = 0.201	.65
Ethnicity (*n*, % white, *n*, % black)	13 (41.9%), 12 (38.7%)	28 (57.1%), 17 (34.7%)	χ^2^(2) = 1.308	.55
CNR event				
Clozapine dose at CNR event (mean, SD)	328.4 (130.2)	375.0 (136.9)	t(71) = 1.449	.08
Leukopenia (*n*, %)	9 (28.1%)	21 (44.7%)	χ^2^(1) = 2.215	.14
Neutropenia (*n*, %)	28 (87.5%)	40 (85.1%)	χ^2^(1) = 0.091	.76
Lymphopenia (*n*, %)	4 (12.5%)	9 (19.1%)	χ^2^(1) = 0.612	.43
Agranulocytosis (*n*, %)	1 (3.1%)	3 (6.1%)	χ^2^(1) = 0.370	1.00
*Medications at CNR status*				
Concomitant antipsychotic (*n*, %)	2 (6.9%)	13 (29.5%)	χ^2^(1) = 5.492	.02^*^
Concomitant valproate (*n*, %)	10 (34.5%)	20 (45.5%)	χ^2^(1) = 0.869	.35
Concomitant other neutropenia-risk drugs^a^ (*n*, %)	1 (3.7%)	5 (11.1%)	χ^2^(1) = 1.212	.40
Concomitant lithium (*n*, %)	2 (6.9%)	3 (6.8%)	χ^2^(1) = 0.000	1.00
*Rechallenge*				
Time to rechallenge, years (mean, SD)	5.1 (6.6)	2.0 (3.5)	t(42.3) = −2.445	.009^*^^*^
Time to rechallenge, years (median, IQR)	1.5 (9.0)	0.32 (1.65)	U = 1056.0	.009^*^^*^
Positive BEN status prior to rechallenge (*n*, %)	14 (46.7%)	14 (29.2%)	χ^2^(1) = 2.457	.12
Follow-up after successful rechallenge, years (mean, SD)	2.5 (3.2)	2.5 (2.2)	t(79) = 0.044	1.00
Follow-up after successful rechallenge, years (median, IQR)	1.3 (3.7)	2.0 (2.5)	U = 670.0	.271
Unsuccessful Rechallenge (re-CNR) (*n*, %)	5 (15.6%)	5 (10.2%)	χ^2^(1) = 0.556	.50
Borderline Rechallenge Outcome^b^	1 (3.1%)	1 (2.0%)	χ^2^(1) = 0.094	1.00

^*^ *P* value < .05

^**^ *P* value < .01

a Medications include lamotrigine (2), lamotrigine and gliclazide (1), gliclazide (1), carbamazepine (1), and mesalazine (1). Abbreviations: CNR, clozapine non-rechallengeable; IQR,

b Clozapine cessation due to hematological considerations, though not formally meeting the criteria for CNR.

**Table 3:** Comparison of Successful and Unsuccessful Rechallenges

	Successful rechallenge n = 69	Unsuccessful rechallenge n = 10	t/χ^2^/U	*P* value
Demographics				
Age at CNR event (mean, SD)	36.3 (13.1)	41.9 (13.1)	t(77) = 1.273	.21
Gender (*n* male, % male)	45, 65.2%	7, 70.0%	χ^2^(1) = 0.089	1.00
Ethnicity (*n*, % white, *n*, % black)	33 (48.5%), 24 (39.7%)	7 (70.0%), 1 (10.0%)	χ^2^(2) = 4.929	.09
First CNR event				
Time to CNR event, years (mean, SD)	4.2 (8.3)	4.7 (5.9)	t(77) = −0.241	.81
Time to CNR event, years (median, IQR)	1.7 (8.7)	1.2 (3.4)	U = 358.5	.84
Leukopenia (*n*, %)	25 (36.8%)	5 (55.6%)	χ^2^(1) = 1.180	.30
Neutropenia (*n*, %)	60 (88.2%)	6 (66.7%)	χ^2^(1) = 3.020	.11
Lymphopenia (*n*, %)	11 (16.2%)	2 (22.2%)	χ^2^(1) = 0.207	.64
Agranulocytosis (*n*, %)	3 (4.4%)	1 (11.1%)	χ^2^(1) = 0.580	.43
Alternative explanations for red results (*n*, %)	9 (13.0%)	1 (10.0%)	χ^2^(1) = 0.364	1.00
*Medications at CNR status*				
Concomitant antipsychotic (*n*, %)	12 (19.7%)	3 (30.0%)	χ^2^(1) = 0.550	.43
Concomitant Valproate (*n*, %)	25 (41.0%)	4 (40.0%)	χ^2^(1) = 0.003	1.00
Concomitant other neutropenia-risk drugs^a^ (*n*,%)	4 (6.7%)	1 (10.0%)	χ^2^(1) = 0.144	.55
Concomitant Lithium (*n*, %)	3 (4.9%)	2 (20.0%)	χ^2^(1) = 2.985	.14
*Rechallenge*				
Time to rechallenge, years (mean, SD)	3.6 (5.5)	0.8 (0.7)	t(76.6) = −4.102	.001^*^^*^
Time to rechallenge, years (median, IQR)	0.9 (5.7)	0.6 (1.1)	U = 385.5	.55
Positive BEN status prior to rechallenge (*n*, %)	25 (37.3%)	2 (22.2%)	χ^2^(1) = 0.789	.48
Concomitant antipsychotic (*n*, %)	6 (24.0%)	3 (33.3%)	χ^2^(1) = 0.296	.67
Concomitant Valproate (*n*, %)	7 (28.0%)	1 (11.1%)	χ^2^(1) = 1.049	.40
Concomitant other neutropenia-risk drugs (*n*, %)^b^	12 (19.0%)	2 (20.0%)	χ^2^(1) = 0.005	1.00
Concomitant Lithium (*n*, %)	10 (40.0%)	3 (33.3%)	χ^2^(1) = 0.125	1.00
Concomitant G-CSF (*n*, %)^a^	0 (0.0%)	4 (5.8%)	χ^2^(1) = 0.611	1.00

Abbreviations: CNR, clozapine non-rechallengable; G-CSF, granulocyte colony-stimulating factor

^*^ *P* value < .05

^**^ *P* value < .01

a Medications include lamotrigine (2), lamotrigine and gliclazide (1), gliclazide (1), and mesalazine (1)

b Medications include lamotrigine (9), lamotrigine and gliclazide (1), gliclazide (2), quinine (1), and mirtazapine (1)

The 2 patients that stopped using clozapine following clinical decisions due to abnormal blood results though not fulfilling CNR criteria were excluded from this analysis.

To exploratively estimate the IRR of BD that are highly likely to be clozapine-induced between the early and late SBD groups, we accounted for the different rechallenge rates between these groups (54.2% vs 69.0%, respectively). This discrepancy is significant, as a lower rechallenge rate in the early group results in fewer opportunities to determine whether the BD was more likely to be clozapine-induced and may obscure a different proportion of clozapine-induced BD. To address this issue, the rechallenge failure rate (the proportion of failed rechallenges out of all rechallenges attempted - 15.6% vs 10.2% in the early and late SBD groups, respectively) was applied to the entire population of each group, including those who were not rechallenged. By applying these failure rates to the entire groups, including non-rechallenged patients, the number of rechallenge-defined clozapine-induced BD cases was estimated to be 9.2 in the early SBD group and 7.2 in the late SBD group. Based on these adjusted estimates, the incidence rate of rechallenge-defined clozapine-induced BD was calculated to be 0.87% per year in the early group and 0.05% per year in the late group, resulting in an exploratory IRR of 15.9. To address potential selection bias, wherein rechallenge attempts may have been more frequently performed for cases more likely to be incidental or non-clozapine-related cases, we conducted a worst-case bounds analysis. This analysis included 2 extreme scenarios, assuming all unchallenged cases were rechallenge-defined clozapine-induced BD, and assuming none of them were clozapine-induced. Under moderate assumptions, the estimated IRR ranged from 12.5 to 14.8. In the extreme scenarios, the IRR varied from 2.3 to 79.8. To note, while the IRR calculation disregards the borderline outcome cases, their 2 possible classifications (either as successful or unsuccessful) are included in the worst-case bounds analysis.

Among the 10 patients who had their CNR status reinstated due to a failed rechallenge (ie, occurrence of a second BD event), 7 (70%) underwent an additional; second rechallenge (meaning, a third clozapine trial). Five of these patients were in the late SBD group, while 2 were in the early SBD group. This pattern may suggest that clinicians were more inclined to attribute SBD to non-clozapine causes in the late SBD group. All 7 patients undergoing a second rechallenge had to discontinue clozapine due to the emergence of a third BD event (second failed rechallenge). Among the 17 failed rechallenge, 10 after the first attempt and 7 after the second, 16 cases (94.1%) lasted shorter than the preceding clozapine trial. Notably, all unsuccessful second rechallenges failed within 60 days of clozapine initiation. The timeframe for repeated CNR events is detailed in the supplemental material (Table S4).

### Agranulocytosis

Seven patients were added to the CNRD due to agranulocytosis. Three of them developed SBD in the “late” period (2-22 years), all of whom were later successfully rechallenged. The other 4 presented with agranulocytosis within the early SBD window (13-154 days), and 3 were not rechallenged. The early SBD patient rechallenged developed a second agranulocytosis and was reassigned with a CNR status.

A detailed breakdown of events by severity per time period is provided in the supplementary material.

### Use of Granulocyte Colony-stimulating Factor

Five patients rechallenged (6.2%) received granulocyte colony-stimulating factor (GCSF) either as per-needed based on the results of their blood count, or as a routine medication during clozapine initiation. None of the patients had CNR status reinstated, though 1 patient stopped using clozapine due to repeated borderline results, necessitating multiple GCSF treatments, which led to a clinical decision to discontinue clozapine.

### Alternative Explanations for Red Results

Among the patients who developed SBD leading to CNR status, 16 had clinical circumstances that may provide alternative explanations, as noted in their clinical records at the time of the event (chemotherapy *n* = 2, infection *n* = 13, and pernicious anemia *n* = 1). None of these patients developed agranulocytosis. Of these 16 patients, 10 underwent rechallenge, including 2 with early-onset BD and 8 with late-onset BD. Nine of these patients had a successful rechallenge, while 1 developed another BD and regained CNR status.

## Discussion

Our findings align with previous evidence that SBD rate is highest during the first year of clozapine treatment.^16–18^^,^^33^ This difference is unlikely to be solely due to detection bias from more frequent blood tests in the early treatment period, as the IRR (10.4) is more than twice the expected value (4.3 weeks per month) if it were solely dependent on monitoring frequency. However, increased early surveillance may still contribute to an inflated detection rate in the early period and should be considered when interpreting these findings. Early and late SBDs were similar in most aspects, differing in parameters of age at SBD event, clozapine dose, and concomitant antipsychotic use. Although age has been proposed as a risk factor for neutropenia,^31^ the difference here likely reflects the longer treatment duration in the late group (mean difference 7.2 years), as both groups had similar ages at clozapine initiation. The higher clozapine dose in the late group likely reflects dose escalation over time, especially in early treatment weeks when most SBDs occurred. Previous studies have not supported a clear relationship between clozapine plasma levels and neutropenia risk.^45^ The greater prevalence of concomitant antipsychotics among late SBD patients may have several explanations. These patients were treated with clozapine for longer, increasing the likelihood of augmentation following partial response (while during the initial treatment period, a “wait-and-see” approach is common). Alternatively, clozapine augmentation may be an independent risk factor for neutropenia.^3^^,^^19^^,^^46^ Another possible explanation is that clozapine-induced BD may be multifactorial, with various contributing factors combining to trigger a neutropenic event. For example, co-administration of valproate has been associated with increased risk of neutropenia. However, our findings highlight that time since clozapine initiation remains a stronger factor than co-prescription. Moreover, if neutropenia occurs only in the presence of valproate, it can be argued that it may not be truly clozapine-induced, supporting continued clozapine use, with adjustment of adjunctive treatments.^47^

Rechallenge failure rates were similar in both early and late SBD groups (15.6% and 10.2%, respectively), as well as in second rechallenge attempts (third clozapine initiation, 100% failures in both groups). However, the exploratory IRR estimate of BD event, more likely to be clozapine-induced BD (15.9), as defined by failed rechallenge, remained markedly higher in the early SBD group even under the most extreme assumptions. This estimate, however, should be interpreted with caution, as it relies on assumptions about the generalizability of failure rates from the rechallenged subgroup. Consistent with previous literature, recurrent neutropenia tended to emerge more quickly than the initial neutropenic event.^27^ Rechallenge failures in both groups occurred within a short timeframe, aligning with the timing definitions of an early BD event. Overall, the high rate of successful rechallenge strongly suggests that most SBDs were not clozapine-induced. Although clozapine was discontinued in all 130 patients, roughly 6 out of 7 SBD cases were unnecessarily attributed to the drug, leading to cessation and increased risk of psychotic deterioration without justification.

It has been stipulated before that early and late SBD events are distinct entities, with early SBD more likely to be clozapine-induced and late SBD more likely reflect an incidental or non-clozapine-related cause.^48^ Our study results could not provide support to this hypothesis. Instead, we found no significant differences in rechallenge success and no clinical differences that would justify different management approaches. Furthermore, the results suggest that not only are most late-onset SBD events unrelated to clozapine, but most early-onset events are not clozapine-induced as well. Additional support for that notion is that rechallenge failures clustered early after re-introduction, regardless of the initial SBD timing, in agreement with previous reports.^23^^,^^26^^,^^27^ All failed third rechallenges also failed at the same early stage, even after years of prior clozapine use, strongly suggesting that BD that is highly likely to be clozapine-induced can occur even after prolonged treatment prior to the initial BD event. However, the literature also includes cases of successful re-rechallenges after a previous failed rechallenge.^34^^,^^49^

Our study found that 75% (3 out of 4) of patients who were rechallenged after agranulocytosis successfully resumed clozapine treatment, a markedly higher success rate than previously reported in the literature.^50^ Notably, all three patients who were successfully rechallenged following agranulocytosis belonged to the late BD group, while the single unsuccessful rechallenge occurred in a patient from the early BD group. Three additional patients who developed agranulocytosis and belonged to the early BD group were not rechallenged. Although statistical inferences are limited by the small sample size, this pattern may imply that the biological implications and prognosis of agranulocytosis could vary depending on whether BD onset is early or late. Furthermore, in our study, only 1 out of the 10 failed rechallenges that are therefore deemed as highly likely clozapine-induced experienced agranulocytosis. This notion is not concordant with previous literature, suggesting that clozapine-induced BD occurs in a distinctive pattern of rapidly declining blood counts, reaching agranulocytosis.^20^^,^^21^ A possible explanation might stem from an early detection and rapid cessation, preventing deterioration to severe neutropenia. This pattern might have been further clarified by 3 additional cases of agranulocytosis in our cohort that occurred early in treatment; however, these patients were not rechallenged. An important consideration arises from the finding that most failed rechallenges followed a mild BD event. As mild events is generally considered to carry a low risk of infection, it is worth considering whether continued clozapine treatment in these cases might not have jeopardized patient safety and whether these events may have resolved spontaneously without escalating to a more severe BD.

Time to rechallenge was significantly longer in the early SBD group, likely reflecting clinician concern about possible true clozapine-induced BD. However, given the high success rates in both groups, early SBD should not preclude rechallenge attempts. Notably, patients with early SBD were less likely to undergo rechallenge, raising the possibility of selection bias and potentially underestimating the true rate of clozapine-induced BD in that group. As a result, it is difficult to draw definitive conclusions.

A significant and unexpected finding was that the successful rechallenges occurred after a mean interval of 3.6 years, whereas the unsuccessful rechallenges followed an interval of 0.8 years. A possible interpretation is that the immune system remains hypersensitized to clozapine after a BD but that this effect diminishes with time. However, this hypothesis should be approached with caution, both on theoretical grounds and based on our data. From a theoretical standpoint, time-dependent changes in immune sensitivity have been described in certain drug-induced hypersensitivity reactions, such as those associated with penicillin.^51^ However, in some cases, hypersensitivity may persist for years.^52^ While the precise mechanism underlying clozapine-induced BD remains unknown, the current evidence does not strongly support a hypersensitivity-based process, making time-limited reactivity less likely. From a data perspective, although all 10 failed rechallenges occurred within 2.1 years of the neutropenic event, a substantial proportion (46 cases, 66.7%) of successful rechallenges also took place within a similar post-BD interval. An additional 14 successful rechallenges (20.3%) occurred 3-10 years post-BD, and 9 (13.0%) were rechallenged after more than a decade. While these data suggest an association between longer intervals and successful rechallenge, the overlap in these intervals complicates any definitive conclusions about time as a determining factor. Given the non-normal distribution of the data (Shapiro–Wilk W = 0.662, *P* < .001) and the non-significant Mann–Whitney *U* test, which does not assume normality, the observed difference may not be as robust as it initially appears. Further studies with greater power are needed to clarify the role of rechallenge timing in its success.

Our study has several limitations. The comparisons between early- and late-onset SBD, as well as between successful and unsuccessful rechallenges, were restricted by data availability. Additional parameters that may be significant—such as treatment adherence (whether patients took clozapine as prescribed), illicit drug use, and nutritional deficiencies—could not be accounted for. Another limitation concerns the generalizability of findings in the rechallenge group, as it remains uncertain whether they apply to all patients who develop SBD. Our data provide limited insight into the selection criteria for rechallenge, but it is likely that some selection took place in favor of patients judged to have a higher chance of success, non-hematological clinical need (such as a high burden of psychotic symptoms), or based on the timing of the BD event. For example, all three patients with late-onset agranulocytosis were rechallenged, whereas only 1 of 4 patients with early-onset agranulocytosis was rechallenged. This decision may be influenced by patient characteristics or clinical factors not accounted for in our analysis, which may partially explain the relatively high rechallenge success rate observed in this study compared to lower success rates reported elsewhere,^50^ though not in all.^53^ This potential bias should be considered when interpreting the results of this study and in assessing their generalizability. A notable example is seen in patients who developed agranulocytosis: only 1 of 4 cases in the early group underwent rechallenge, whereas all 3 late-onset agranulocytosis cases were rechallenged. This difference is particularly important given that all patients in the late group were successfully rechallenged, while the single rechallenged agranulocytosis patient in the early group experienced a second BD event. Additionally, although our sample includes one of the largest rechallenge cohorts described to date, repeated rechallenges remain rare, limiting the statistical power to analyze this subgroup.

This study did not account for longitudinal changes in co-prescribed medications, such as valproate or lithium, between the initial BD event and the rechallenge attempt. As these agents may influence neutropenia risk, they could also affect the likelihood of rechallenge success. Future studies should explore their dynamic role over time in relation to rechallenge outcomes.

While rechallenge outcome was used in this study as a clinical proxy true clozapine-induced BD, this approach has inherent limitations. Some rechallenge “failures” may not result from a pharmacological or immunological reaction to clozapine itself, but rather from persistent or intermittent low neutrophil counts that trigger cessation under current monitoring thresholds. This concern is particularly relevant in cases where the initial BD event was mild, a pattern observed in the majority of failed rechallenges in our cohort. However, this explanation may be less applicable to late-onset BD events, the primary focus of this study, as these patients had tolerated clozapine uneventfully for years, at times, prior to the emergence of blood abnormalities. This temporal dissociation supports the interpretation that late-onset cases, when rechallenge fails, are more likely to reflect acquired clozapine sensitivity. Whether this acquired clozapine sensitivity carries clinical risk for infection or progression to more severe BD remains an open question and warrants further study. It should also be noted that 2 rechallenge cases were discontinued due to hematological concerns without meeting formal CNR criteria, classified as “borderline outcomes” and excluded from the binary outcome analysis. While their exclusion avoided misclassification, it introduces a gray zone that reflects real-world complexity in clinical decision-making and limits the precision of outcome categorization.

Additional methodological limitations may affect interpretation of the incidence data. The rates of SBD during clozapine treatment in our study should be interpreted with caution. The start dates were estimated using an algorithm, and in some cases may have been inaccurate.^43^ Additionally, the SLaM NHS Foundation Trust houses the National Psychosis Unit (NPU), a specialized center for clozapine therapy that frequently receives referrals from other UK regions for clozapine rechallenge, thus contributing patients to our dataset. This focus may lead to an increased representation of clozapine-associated SBD cases, potentially inflating incidence rates in our cohort. In addition, our cohort includes a high rate of valproate co-prescription, which may confound the observed BD incidence and rechallenge outcomes due to valproate’s suspected role in inducing or augmenting neutropenia. Moreover, these rates may reflect local clinical practices and may not be generalizable. Notably, despite this possible overrepresentation, there were no deaths attributable to clozapine-induced BD.

Although our study suggests that BD events, highly likely to be clozapine-induced, may occur even years after initiating clozapine therapy, it is essential to view this finding in a broader context.^37^^,^^54^ First, the incidence rate remains extremely low: only 5 cases of BD truly attributed to clozapine in our cohort (based on failed clozapine rechallenge) occurred after 6 months of treatment. This represents an incident rate of 0.05% per year and affects approximately 1 in every 328 individuals who continued clozapine beyond 6 months. Some studies indicate that this low incidence rate may not significantly differ from the baseline rate of BD with other antipsychotics,^46^^,^^54^ although findings are mixed.^37^ Furthermore, as research suggests that mild neutropenia often resolves spontaneously, it frequently prompts unnecessary clozapine discontinuation under current guidelines. As a result, the net clinical impact of continuous lifelong blood monitoring is questionable, as the associated reduced prescribing, premature discontinuation, and possible clinical deterioration may ultimately result in greater harm than the rare missed late-onset BD case due to less frequent monitoring. It may also be argued that any monitoring frequency less than weekly risks missing a rapidly developing agranulocytosis, so a more logical regime would monitor weekly during the high-risk phase (eg, for 1 year) and then be discontinued altogether. While a risk-stratified monitoring strategy, tailored to individual risk, would offer the correct balance, current scientific data is insufficient to support implementation of such a model in routine practice, and further research is needed.

## Conclusions

The results of this study suggest that although the incidence of blood dyscrasia decreases over time, such events may occur years after clozapine initiation and recur upon rechallenge. Our study found no significant demographic or clinical differences between BD occurring before or after 6 months of therapy, nor between patients successfully rechallenged and those who experienced recurrent episodes of blood dyscrasia. While this study highlights the ongoing but small risk of clozapine-induced blood dyscrasia even after years of treatment, potentially supporting the need for continuous blood monitoring, this finding should be considered within a broader context: the low incidence combined with evidence suggesting that other antipsychotics may lead to blood dyscrasia at similar rates yet do not require monitoring. Overall, this underscores the need to reevaluate current guidelines to better balance the harms of mandatory lifelong monthly blood monitoring, such as unnecessary clozapine discontinuations and reduced adherence, against the risk of late-onset blood dyscrasia that is truly clozapine-induced.

## Supplementary Material

Supplementary_materials_sbaf148

## Data Availability

The data used in this study is available in the CRIS system, as well as the database created by this study. However, CRIS data is available to researchers at SLaM only, due to patients’ confidentiality. Access to it requires authorization from SLaM BRC (Biomedical Research Centre).
