# Supplementary material for: Late-Onset Neutropenia in Clozapine Users: Unrelated or Drug-Induced? A Case-Registry Analysis of Incidence, Characteristics, and Rechallenge Attempts
Source: Schizophr Bull. 2025 Aug 26;52(4):sbaf148. doi: 10.1093/schbul/sbaf148 (PMC13391638; doi:10.1093/schbul/sbaf148)
Supplement: Supplementary_materials_sbaf148 [file supplementary_materials_sbaf148.docx]

**Supplemental Material**

Table A. Breakdown of blood-dyscrasia events by timeframes and severity of event

| Severity of blood dyscrasia | < 6 months n (%) | 6-12 months n (%) | 12-24 months n (%) | >24 months n (%) | Total |
| --- | --- | --- | --- | --- | --- |
| Mild | 44 (42.7%) | 9 (8.7%) | 9 (8.7%) | 41 (39.8%) | 103 (100.0%) |
| Moderate | 11 (73.3%) | 0 (0.0%) | 0 (0.0%) | 4 (26.6%) | 15 (100.0%) |
| Severe | 2 (50%) | 0 (0.0%) | 1 (25%) | 1 (25%) | 4 (100.0%) |
| Unknown | 2 (25.0%) | 0 (0.0%) | 2 (25.0%) | 4 (50.0%) | 8 (100.0%) |
|  |  |  |  |  |  |
| Total events per timeframe | 59 (45.4%) | 9 (6.9%) | 12 (9.2%) | 50 (38.5) | 130 (100.0%) |
| Total non-mild events per timeframe | 13 (68.4%) | 0 (0.0%) | 1 (5.3%) | 5 (26.3%) | 19 (100.0%) |

Table B. Comparison of patients developing apparent blood dyscrasias who were not rechallenged versus those who were rechallenge on clozapine.

|  | | | CNR patients not rechallenged  N=49 | CNR patients rechallenged  N=81 | t / χ²/ U | p Value |
| --- | --- | --- | --- | --- | --- | --- |
| Demographics | | |  |  |  |  |
|  | Age at CNR status (mean, SD) | | 43.2 (15.0) | 37.1 (13.0) | t(128)=2.448 | 0.02* |
|  | Gender (n, % male) | | 29 (59.2%) | 53 (65.4%) | χ²(1)=0.512 | 0.47 |
|  | Ethnicity (n, % white, n, % black) | | 18 (36.7%),  24 (49.0%) | 41 (51.2%),  29 (36.3%) | χ²(1)=2.686 | 0.44 |
| CNR Event | | |  |  |  |  |
|  | Time to CNR event, years (mean, SD) | | 3.2 (5.5) | 4.7 (6.1) | t(128)=-1.310 | 0.19 |
|  | Time to CNR event, years (median, IQR) | | 0.4 (4.4) | 1.7 (7.3) | U=2439.500 | 0.03* |
|  | Clozapine dose at CNR event (mean, SD) | | 302.8 (125.9) | 356.5 (135.3) | t(120)=-2.140 | 0.03* |
|  | Leukopenia (n, %) | | 19 (40.5%) | 30 (38.0%) | χ²(1)=0.074 | 0.79 |
|  | Neutropenia (n, %) | | 45 (93.8%) | 68 (86.1%) | χ²(1)=1.793 | 0.18 |
|  | Lymphopenia (n, %) | | 7 (14.6%) | 13 (16.5%) | χ²(1)=0.079 | 0.78 |
|  | Agranulocytosis (n, %) | | 3 (6.3%) | 4 (4.9%) | χ²(1)=0.101 | 0.71 |
|  | Early Blood Dyscrasia (n, %) | | 27 (55.1%) | 32 (39.5%) | χ²(1)=2.996 | 0.08 |
|  | Concomitant medications at CNR event | |  |  |  |  |
|  | | Concomitant antipsychotic (n, %) | 11 (22.4%) | 15 (20.5%) | χ²(1)=0.063 | 0.80 |
|  | | Concomitant Valproate (n, %) | 21 (42.9) | 30 (41.1%) | χ²(1)=0.037 | 0.85 |
|  | | Concomitant other neutropenia-risk drugs (n, %) ^a^ | 6 (8.3%) | 7 (4.3%) | χ²(1)=1.077 | 0.37 |
|  | | Concomitant Lithium (n, %) | 5 (10.2%) | 5 (6.8%) | χ²(1)=0.439 | 0.52 |

* p Value < 0.05; ** p Value < 0.01. ^a^ Medications include lamotrigine (5), lamotrigine and gliclazide (1), gliclazide (2), spironolactone (2), carbamazepine (1) and mesalazine (1). CNR – Clozapine Non-Rechallengeable

Table C. Comparison of patients successfully rechallenged after early onset apparent blood dyscrasias versus those who were successfully rechallenged after early onset apparent blood dyscrasias.

|  | | | Successfully Rechallenge  After early ABD  N=26 | Successfully Rechallenge  After late ABD  N=43 | t / χ²/ U | p Value |
| --- | --- | --- | --- | --- | --- | --- |
| Demographics | | |  |  |  |  |
|  | Age at CNR event (mean, SD) | | 29.0 (9.9) | 40.7 (12.8) | t(67)=3.991 | 0.001** |
|  | Age at clozapine initiation | | 28.0 (9.9) | 32.7 (9.9) | t(67)=1.912 | 0.06 |
|  | Gender (n, % male) | | 17 (65.4%) | 28 (65.1%) | χ²(1)=0.001 | 0.98 |
|  | Ethnicity (n, % white, n, % black) | | 9 (36.0%), 12 (48.0%) | 24 (55.8%), 15 (34.9%) | χ²(1)=4.534 | 0.17 |
| First CNR Event | | |  |  |  |  |
|  | Time to CNR event, years (mean, SD) | | 0.2 (0.15) | 7.5 (6.0) | t(42.1)=7.905 | 0.001** |
|  | Time to CNR event, years (median, IQR) | | 0.20 (0.24) | 6.0 (12.0) | U=0.000 | 0.001** |
|  | Clozapine dose at CNR event (mean, SD) | | 306.5 (127.7) | 377.0 (145.5) | t(59)=1.917 | 0.06* |
|  | Leukopenia (n, %) | | 7 (26.9%) | 18 (42.9%) | χ²(1)=1.754 | 0.185 |
|  | Neutropenia (n, %) | | 22 (84.6%) | 38 (90.5%) | χ²(1)=0.531 | 0.47 |
|  | Lymphopenia (n, %) | | 4 (15.4%) | 7 (16.7%) | χ²(1)=0.019 | 1.00 |
|  | Agranulocytosis (n, %) | | 0 (0.0%) | 3 (7.0%) | χ²(1)=1.896 | 0.29 |
|  | Medications at CNR status | |  |  |  |  |
|  | | Concomitant antipsychotic (n, %) | 2 (8.7%) | 10 (26.3%) | χ²(1)=2.815 | 0.11 |
|  | | Concomitant Valproate (n, %) | 7 (30.4%) | 18 (47.4%) | χ²(1)=1.699 | 0.19 |
|  | | Concomitant other neutropenia-risk drugs (n, %) ^a^ | 0 (0.0%) | 4 (10.3%) | χ²(1)=2.308 | 0.29 |
|  | | Concomitant Lithium (n, %) | 2 (8.7%) | 1 (2.6%) | χ²(1)=1.127 | 0.55 |
| Rechallenge | | |  |  |  |  |
|  | Time to rechallenge, years (mean, SD) | | 6.0 (7.1) | 2.2 (3.7) | t(33.2)=-2.518 | 0.017* |
|  | Time to rechallenge, years (median, IQR) | | 1.7 (10.9) | 0.52 (2.9) | U = 739.0 | 0.026* |
|  | BEN status prior to rechallenge (n, %) ^a^ | | 13 (52..0%) | 12 (28.6%) | χ²(1)=3.678 | 0.055 |
|  | Concomitant antipsychotic (n, %) ^a^ | | 1 (20.0%) | 5 (25.0%) | χ²(1)=0.055 | 1.00 |
|  | Concomitant Valproate (n, %) ^a^ | | 2 (40.0%) | 5 (25.0%) | χ²(1)=0.446 | 0.60 |
|  | Concomitant other neutropenia-risk drugs (n, %) ^b^ | | 5 (20.8%) | 7 (17.9%) | χ²(1)=0.080 | 1.00 |
|  | Concomitant Lithium (n, %) ^b^ | | 2 (40.0%) | 8 (40.0%) | χ²(1)=0.000 | 1.00 |
|  | Follow-up of successful rechallenge, years (mean, SD) | | 3.0 (3.3) | 2.8 (2.2) | t(67)=-0.290 | 0.77 |
|  | Follow-up of successful rechallenge, years (median, IQR) | | 1.3 (3.7) | 2.0 (2.5) | U = 504.0 | 0.50 |

* p Value < 0.05; ** p Value < 0.01; ^a^ Medication includes lamotrigine (2), gliclazide (1), mesalamine (1); ^b^ Data was available for 25 patients only: 5 om the early BD group and 20 in the late BD group.

Table D. Time frame to repeated CNR events.

| Patient number | Days to first CNR | Days to second CNR | Days to third CNR |
| --- | --- | --- | --- |
| 1 | 10046 | 41 | 58 |
| 2 | 1612 | 45 | 34 |
| 3 | 1269 | 61 | 27 |
| 4 | 1266 | 49 | 32 |
| 5 | 731 | 139 | 20 |
| 6 | 152 | 43 | 26 |
| 7 | 138 | 47 | 33 |
| 8 | 102 | 98 |  |
| 9 | 95 | 33 |  |
| 10 | 57 | 26 |  |

CNR – Clozapine Non-Rechallenge status.
